# Comparison of Activity and Safety of DSPAα1 and Its N-Glycosylation Mutants

**DOI:** 10.3390/life13040985

**Published:** 2023-04-11

**Authors:** Huakang Peng, Nan Wang, Mengqi Wang, Caifeng Yang, Wenfang Guo, Gangqiang Li, Sumei Huang, Di Wei, Dehu Liu

**Affiliations:** 1 Biotechnology Research Institute, Chinese Academy of Agricultural Sciences, Beijing 100081, China; 2 Biotechnology Research Institute, Guangxi Academy of Agricultural Sciences, Nanning 530007, China

**Keywords:** N-glycosylation sites, plasminogen activator, fibrin selectivity, DSPAα1, rat, drug safety

## Abstract

DSPAα1 is a potent rude thrombolytic protein with high medicative value. DSPAα1 has two natural N-glycan sites (N153Q-S154-S155, N398Q-K399-T400) that may lead to immune responses when administered in vivo. We aimed to study the effect of its N-glycosylation sites on DSPAα1 in vitro and in vivo by mutating these N-glycosylation sites. In this experiment, four single mutants and one double mutant were predicted and expressed in *Pichia pastoris*. When the N398Q-K399-T400 site was mutated, the fibrinolytic activity of the mutant was reduced by 75%. When the N153Q-S154-S155 sites were inactivated as described above, the plasminogen activating activity of its mutant was reduced by 40%, and fibrin selectivity was significantly reduced by 21-fold. The introduction of N-glycosylation on N184-G185-A186T and K368N-S369-S370 also considerably reduced the activity and fibrin selectivity of DSPAα1. The pH tolerance and thermotolerance of all mutants did not change significantly. In vivo experiments also confirmed that N-glycosylation mutations can reduce the safety of DSPAα1, lead to prolonged bleeding time, non-physiological reduction of coagulation factor (α2-AP, PAI) concentration, and increase the risk of irregular bleeding. This study ultimately demonstrated the effect of N-glycosylation mutations on the activity and safety of DSPAα1.

## 1. Introduction

Thromboembolic disease has become a serious health hazard due to the aging of the worldwide population and dramatic lifestyle changes. Some studies have suggested that thromboembolic disease is a complication of COVID-19 [1]. As COVID-19 continues to spread globally, the incidence of the thrombotic disease is also rising sharply [2]. Although the relevant evidence is limited, there is still an urgent need to develop novel thrombolytic drugs. Despite years of research, thrombolytic drugs are still unable to eliminate the problems of low fibrin selectivity and momentary half-life, which bring difficulties to treat patients with embolisms [3,4,5]. The vampire bat plasminogen activator (DSPA) is an anticoagulant found in vampire bat saliva [6]; it activates plasminogen to plasmin, which promotes the dissolution of blood clots and hinders blood clotting [7,8].

DSPA has four forms, with DSPAα1 being the most active and most widely studied of the four forms [9]. DSPAα1 has a long half-life, which prolongs the rescue time of patients, and it can also bind to fibrin without non-specific bleeding, making DSPAα1 one of the most effective thrombosis treatments [10]. Mature DSPAα1 consists of 477 amino acids and contains four protein domains, namely the Finger region (F), the EGF region (EGF), the Kringle1 region (K1), and the serine protease region (P) [9]. Tissue plasminogen activator (t-PA) is a human plasminogen activator and the most studied and modified plasminogen activator, and its related mutants and isomers are widely used in clinical practice. DSPAα1 and t-PA are highly homologous. The main difference is that DSPAα1 does not have the Kringle 2 region and plasmin-sensitive cleavage site compared with t-PA [11] the dependence of DSPAα1 and its selectivity for fibrin is higher [12,13,14]. When fibrin is present as a cofactor, the catalytic activity of DSPAα1 increases by 10^5^-fold [15,16], while this increases by only 550-fold for t-PA [17]. Animal experiments have also proven that DSPAα1 has a faster thrombolysis rate, longer thrombolysis time, and less non-specific thrombolysis reaction compared with alteplase (rt-PA) [18,19]. To date, DSPAα1 has been investigated for its pharmaceutical potential and had entered Phase III clinical trial testing [20], but these trials were rejected in 2019 because of poor immune responses and treatment effects.

Glycosylation of proteins refers to the addition of glycan chains to proteins under the control of various enzymes [21]. N-glycosylation is the most ordinary and most studied form of glycosylation in eukaryotes [22]. N-glycosylation usually occurs on Asn in the consensus sequence Asn-X-Ser/Thr (X can be any amino acid except Pro). N-glycosylation can increase the solubility of glycoprotein and reduce the aggregation of glycoprotein, which will result in clearance and low activity [23]. N-glycosylation can also guide the folding and transport of proteins, endow or alter the distinctive activities of glycoproteins, and make the modified proteins more effective at participating in various essential physiological functions [24]. However, N-glycosylation is a problem that must be considered for use in pharmaceutical proteins. The N-glycan chain brings immunogenicity to the protein for administration [25]. The N-glycosylation of *Pichia pastoris* is a high-mannose type, while mammalian cells are a complex type. High-mannose N-glycans typically contain between two and six Mannose-residues (Man-residues) which may intensify the immune response attached to the pentasaccharide core while mammalian structures comprise a Man-residues [26]. 

Thankfully, N-glycoengineered *Pichia pastoris* have been used to produce therapeutic glycoproteins candidates with a completely reengineered and humanized N-glycosylation pathway. However, in addition to enabling the production of heterologous proteins with human N-glycosylation patterns, N-glycoengineering also changed the glycan structures of all endogenous glycoproteins within the Pichia host cell [27]. Therefore, the simplest way to prevent immune reactions is to use the mutations without N-glycosylation. However, deglycosylation mutations may affect protein activity [28]. 

DSPAα1 has two potential native N-glycosylation sites and several potential N-glycosylation sites, the same as DSPAα2. The N-glycosylation site of DSPAα2 was studied and the effect of the site on activity was verified [29]. However, we found that the activity and application value of DSPAα1 were higher than those of DSPAα2 as our research deepens. There are more studies and applications on DSPAα1 than DSPAα2. The activity and structure of DSPAα1 are also studied more thoroughly than DSPAα2. DSPAα1 has been expressed in multiple expression systems (COS, BHK, CHO, insect cell, *P. pastoris*, rabbit mammary gland) [11,30,31,32,33], and the activity and structure of DSPAα1 have been thoroughly studied. However, there is a lack of systematic research studies into its N-glycosylation. Although we have verified the absence or increase of N-glycan chains on DSPAα2 alters the activity of DSPAα2 [29], and some studies have shown that different types of N-glycan chains did not significantly change DSPAα1 activity [31,34]. However, we believe that N-glycosylation studies for DSPAα1, a potential therapeutic glycoprotein, are still necessary. Therefore, based on the experimental strategy of DSPAα2, multiple N-glycosylation mutants of DSPAα1 were constructed and expressed in *P. pastoris* GS115 according to N-glycosylation site prediction and homology modeling analysis. We want to be able to compare the activity and stability of DSPAα1 and its mutants. Herein, we have elucidated the effect of N-glycosylation on the biochemical functions of DSPAα1 in vitro and in vivo and we provide a reference for a follow-up study of DSPAα1.

## 2. Methods and Materials

### 2.1. Preparation of Plasmids and Strains

*Escherichia coli* TOP10 (Zoman Bio, Beijing, China) was used for plasmid proliferation with kanamycin (50 μg/mL) and *P. pastoris* GS115 was used for expression. The plasmid *Ppic9k* used was embellished by using the *Gap* promoter and renamed as *Ppic9k-Gap*. The transformed *P. pastoris* clonings were cultivated at 30 °C on RD medium plates (20% D-glucose, 0.5% Amino Acids Blend, 13.4% Yeast nitrogen base with ammonium sulfate without amino acids, 0.02% biotin) containing 0.25 mg/mL geneticin (G418, Solarbio, Beijing, China). The multicopy clones were cultivated in BMGY mediums (1% Yeast extract, 2% Peptone, 100 mM potassium phosphate buffer, pH 6.0, 1.34% yeast nitrogen base without amino acids, 4 × 10^−5^% biotin, and 1% glycerol) containing 1–4 mg/mL G418. Culture was performed in a shaker (200 rpm) at 30 °C, and the growth of strains was surveillanced by detecting the optical density at 600 nm.

### 2.2. Mutagenesis of DSPAα1

The codon sequence was optimized according to the sequenced *DSPAα1* nucleotide sequence (GenBank ID M63987), making the gene sequence more suitable for expression in *P. pastoris*. The optimized cDNA sequence was then integrated into the *Ppic9k-Gap* vector by high-fidelity PCR and homologous recombination to construct *DSPAα1-Ppic9k-Gap*. The recombinant plasmid *DSPAα1-Ppic9K-Gap* harbors *DSPAα1* and a 6*His-tag at the C terminus. Potentially mutagenic N-glycosylation sites were identified using homology alignment and modeling analysis [29]. N-glycosylation site analyzing was conducted using the NetNGlyc 1.0 Server (http://www.cbs.dtu.dk/services/NetNGlyc/, accessed on 10 September 2022). Homologous sequence alignment was performed using Clustal W (https://www.ebi.ac.uk/Tools/msa/clustalo/, accessed on 10 September 2022). The homology-modeled structure of the DSPAα1 was used to predict the biological function of candidate mutation sites by PyMOL [35,36]. The model was predicted by the AlphaFold database (https://alphafold.com/, accessed on 13 September 2022). The mutant plasmids were constructed by site-directed mutagenesis with the plasmid *DSPAα1-Ppic9k-Gap* as a PCR template. The five mutants were QNGlyα1-1 (N153Q-S154-S155), QNGlyα1-2 (N398Q-K399-T400), QNGlyα1 (N153Q-S154-S155\N398Q-K399-T400), ANGlyα1 + 2 (N184-G185-A186T), ANGlyα1 + U (K368N-S369-S370). All the plasmids were transformed into *Escherichia coli* TOP10. Positive clones have emerged on the LB plates supplemented with 50 μg/mL kanamycin. Mutagenic primers were synthesized by Sangon (Shanghai, China) and are summarized in Supplement Table S1.

### 2.3. Expression and Purification of Wild-Type and Mutant DSPAα1

The identified recombinant plasmid was linearized using *Sal* I (NEB, Ipswich, SU, UK) and electrotransformed into *P. pastoris* GS115. The electroporation protocol was carried out according to the *Pichia* expression kit (Invitrogen, Waltham, MA, USA). The transformants that emerged on RD plates supplemented with 0.25 mg/mL G418 were inoculated to YPD medium plates supplemented with 1–4 mg/mL G418 (Solaribo, Beijing, China) and cultured at 28 °C for three days to select multicopy integrants.

The positive clones were screened by genomic PCR and rt-PCR. In order to determine the level of expression of these positive clones, we used genomic qPCR to screen the relative copy numbers of those multicopy clones that can grow on YPD medium plates supplemented with 4 mg/mL G418. The primers were synthesised by Sangon (Shanghai, China) and are summarised in Supplement Table S1.

The total DNA and RNA extraction was performed according to the manufacturer’s guidelines of the DNA and RNA Purification Kit (Zoman Bio, Beijing, China). The genomic PCR and rt-PCR were performed using a PCR SuperMix Kit (Zoman Bio, Beijing, China). The quantitative PCR (qPCR) was performed using a qPCR SuperMix Kit (Zoman Bio, Beijing, China) on a QuantStudio 3 instrument (Thermo Fisher Scientific, Waltham, MA, USA). The actin gene of *P. pastoris* GS115 was the reference gene. The relative copies at the DNA level were calculated by the relative 2^–ΔΔCT^ method [37].

After five days of culture, the cell-free culture supernatant was collected by centrifugation at 4000 rpm for 30 min. Then, the supernatant was concentrated by transferring it to a dialysis bag covered with PEG20000 and 4 °C overnight. The suspension was centrifuged at 4000 rpm for 30 min and then diluted in a 20 mM phosphate buffer solution (pH 7.4). Afterward, his-tagged recombinant enzymes were purified using Ni^2+^ affinity chromatography (Sangon, Shanghai, China). The Bradford rapid protein quantification kit (Zoman Bio, Beijing, China) was used to determine the concentration of proteins, and BSA (1 mg/mL) was used as the standards [38].

### 2.4. SDS-PAGE Analysis

SDS-PAGE analysis was carried out in a 10% (*w*/*v*) polyacrylamide gel. Coomassie blue R-250 (Sigma-Aldrich, St. Louis, MI, USA) and western blot were applied to identify these samples before and after cut by PNGase F (NEB, Ipswich, SU, UK). PNGase F, the best enzyme for removing N-glycan chains from glycoproteins, was used to remove the N-glycan chains. Proteins were stained with Coomassie blue R250 with the medium protein marker (Zoman Bio, Beijing, China) as a standard.

Glycoprotein staining was also applied to identify these samples without PNGase F cleavage to prove that all N-glycosylation sites on the mutants have been N-glycosylated with a glycoprotein staining kit (Invitrogen, Waltham, MA, USA).

### 2.5. Fibrin Assay of DSPAα1 Activity

Fibrinogen, thrombin, and t-PA were purchased from Abclonal (Wuhan, China). Fibrinolytic activity was measured by the modified fibrin assay as described [39]. Gradient dilution standard t-PA is added to the well as samples of different activities. After 12 h, the logarithm of the average diameter is the ordinate coordinate. The logarithm of the activity is the abscissa. The change in the curve between the logarithm of the average fibrinolytic diameter and the logarithm of activity is used to obtain the standard curve. The fibrinolytic activity was computed by evaluating the calculated acreage of the lytic area contrasted to t-PA. Data were displayed as means ± SD (*n* = 3). Mean values were computed by an average of three replicates (*n* = 3). The significant differences between samples were computed by *t*-test.

### 2.6. Kinetics of S-2765 Hydrolysis

The assay volume was 0.15 mL containing 10 nM samples, 100 μg/mL fibrin(ogen) where stated (0.15 units/mL human thrombin in case of fibrin), and 0.01–5 mM S-2765 in assay buffer (50 mM Na_2_CO_3_, 0.1% Tween-80, pH 7.0). Individual assays were performed three times. Hydrolysis of S-2765 by thrombin was not detectable under these conditions. To correct for turbidity due to fibrin formation, △A405-A490/min was monitored. Omitting the plasminogen activator, blanks were also performed in duplicate for every concentration of S-2765. As described above, △A405/min was calculated and converted to [*pNA*]. According to the [*pNA*] standard curve, velocities were plotted against the concentration of S-2765 and analyzed by the Michaelis–Menten equation to obtain the kinetic parameters *K*m, *K*cat, and *K*cat/*K*m [40]. Data were presented as means ± SD (*n* = 3).

### 2.7. Detection of Thermal Stability and Acid-Base Stability

The thermostability was detected by nanoDSF (nano Differential Scanning Fluorimetry). The optimal reaction temperature and pH of DSPAα1 and its N-glycosylated mutations were analyzed by the S-2765 hydrolysis after the enzymes were incubated with 0.01 M PBS in the range of 30–90 °C or pH 3.0–12.0 for 1 h.

### 2.8. Rat Modelling and Drug Delivery

All experiments were performed according to the relevant guidelines and regulations of AEWC (Animal Ethical and Welfare Committee of BRI, CAAS).

After 7 days of culture [20–25 °C, 45–55% relative humidity, Specific pathogen-free (SPF) rat chow], the *Sprague-Dawley* (SD) rats (6–8 weeks old, 230 ± 30 g, half were male, and half were female, SPF) were randomly divided into six groups (eight rats per group). All the groups except Group untreated control (Ck) were induced carotid thrombosis by FeCl_3_-induced carotid artery thrombosis assay after anesthetized as described [41]. 

Drug delivery was performed 60 min after thrombus induction. The tail-vein infusion method was used [42]. Rats were injected with saline, rt-PA, rDSPAα1, QNGlyα1, or ANGlyα1 + 2. All drugs were injected at a concentration of 1 mg/kg and were diluted with isotonic saline to a final volume of 5 mL. A bolus of 2.5 mL of the drug was administered by tail-vein infusion, and the remaining 2.5 mL of the drug was administered within 1 h by the infusion pump.

### 2.9. Thrombus Detection and Hemostatic Factors Analysis

The bleeding time was measured using Gardell’s protocol [43,44]. Blood collection and platelet parameters were performed simultaneously with the determination of bleeding time. See Table 1 for blood sampling time. Platelet parameters were obtained by blood routine examination and the left carotid artery cannula was used for blood sampling. Plasma was derived from centrifugation of blood samples (1500× *g*, 10 min, 4 °C) and kept in EDTA presoak tubes (5 μL/mL of 1 μM D-Phe-Pro-Arg-CH_2_Cl was added; Russo et al., 2012). Fibrinogen (Fbg), plasminogen, α2-antiplasmin factor (α2-AP), PAI, fibrin (ogen) degradation product (FDP), and D-2-mer (D-D) were measured from plasma samples by ELISA kits (enzyme label, Yancheng, China).

After blood samples were collected, rats are sacrificed and the thrombus is removed for Hematoxylin and Eosin (H & E) staining and analysis [45]. Whether recanalization of the thrombus occurred was determined based on the presence of blood outflow. The removed thrombus clots were used to perform the three indicators (dry weight, wet weight, and length of thrombus) of in vitro thrombosis detection and H&E staining, respectively. Rats in group CK were smeared with blood from the left carotid artery. All operations were performed under deep anesthesia. All data were presented as means ± SD (*n* = 8). 

### 2.10. Thrombopathological Biopsy Analysis

Observing H & E staining sections and performing thrombopathological biopsy analysis can more accurately reflect the severity of the thrombus. This study was performed as a blinded randomized trial. For the analysis, score values for P1 (Score of arterial endothelial injury), P2 (Score of thrombus proportion), P3 (Score of cellulose percentage), P4 (Score of red blood cell ratio), P5 (Score of fracture ratio) and P6 (Score of WBC) are indicated. All indexes were scored as 20 points. While the higher the points, the worse the thrombolytic efficiency.

## 3. Results

### 3.1. Prediction of N-Glycosylation Sites and Structural Predictive Analysis

**Figure 1:**
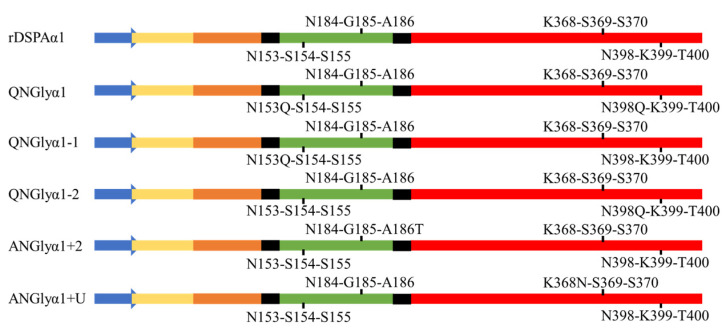
Demonstration of N-glycan sites of DSPAα1 and its mutants. N-glycan sites are marked in the figure. The blue arrow represents the signal peptide of DSPAα1; the yellow rectangle represents the F region; the orange represents the EGF region; the green represents the K1 region; the red represents the P region; the black represents the connections between domains.

**Figure 2:**
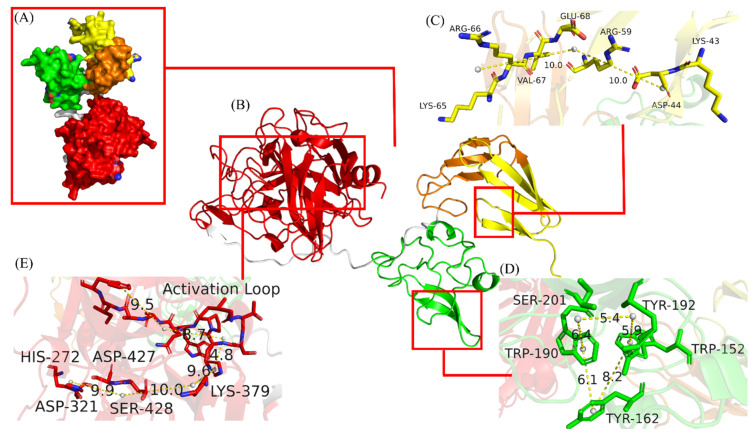
The predicted structure of DSPAα1. This structure was generated using the AlphaFold Protein Structure Database and annotated in PyMOL. (**A**) The solvent-accessible surface of DSPAα1. (**B**) The carton structure of DSPAα1. The yellow helices and sheets represent the F region, the orange represents the EGF region, the green represents the K1 region, and the red represents the P region. (**C**) Detailed view of fibrin selective related sites. (**D**) A clear view of ligand-binding sites. (**E**) Display of the active pocket. The structure of amino acids was represented by sticks of different colors. The yellow dotted lines represent the interaction distance between residues, and the numbers on the dotted line indicate the spatial distance between them.

**Figure 3:**
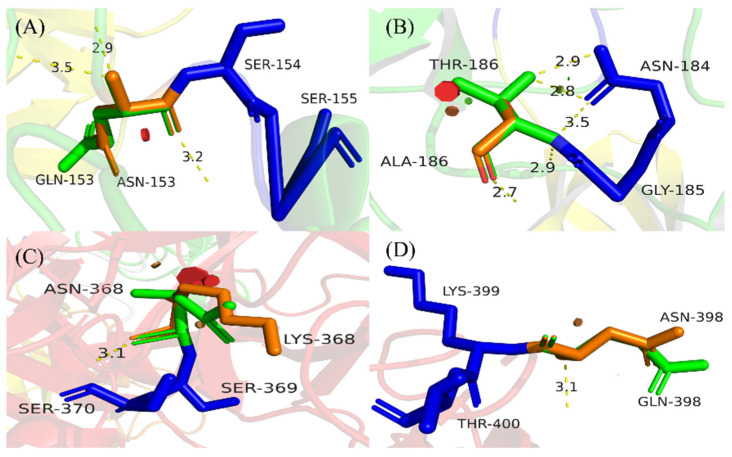
Prediction of all polar neighbor distances at mutation sites before and after mutation. This figure was generated and annotated in PyMOL. (**A**–**D**) Orange sticks represent the wild-type amino acid residue and green sticks represent the mutated amino acid residue. Blue sticks indicate residues linking to the mutated residue. Yellow dotted lines represent all the polar neighbor distances of the site; (**A**) N153Q-S154-S155, (**B**) N184-G185-A186T, (**C**) K368N-S369-S370, (**D**) N398Q-K399-T400.

**Figure 4:**
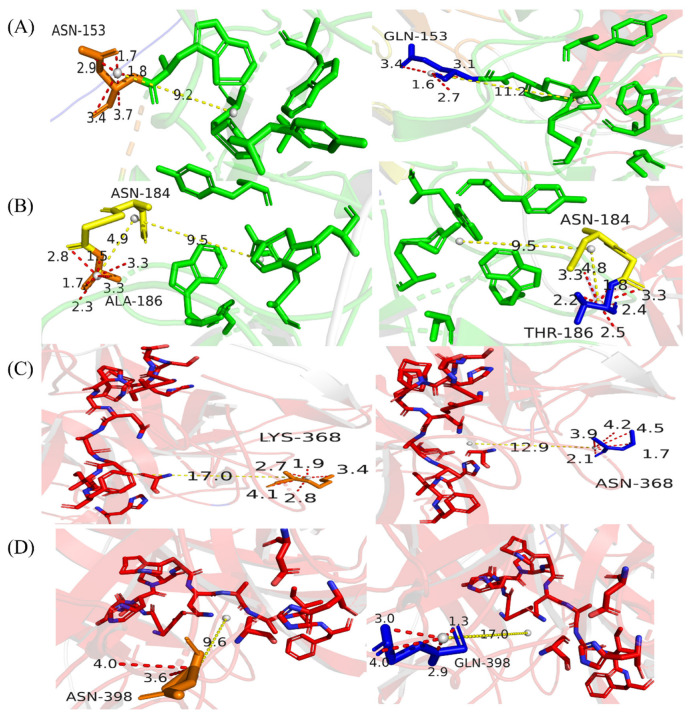
Prediction of spatial distances and adjacent force at N-glycosylation sites before and after mutation. This figure was generated and annotated in PyMOL. (**A**–**D**) Orange sticks represent the wild-type amino acid residue and blue sticks represent the mutated amino acid residue. Yellow sticks indicate residues linking to the mutated residue. The region is indicated by green or red sticks. Yellow dotted lines represent the spatial distances of the site while red dotted lines represent the adjacent forces of the site; (**A**) N153Q-S154-S155, (**B**) N184-G185-A186T, (**C**) K368N-S369-S370, (**D**) N398Q-K399-T400.

We first used NetNGlyc to predict potential N-glycosylation sites in DSPAα1 and identified two N-glycan sites (N153Q-S154-S155, N398Q-K399-T400). DSPAα1 has multiple homologous proteins (u-PA, t-PA, DSPAα2). We compared the DSPAα1 protein sequence to its homologous proteins and identified another two potential N-glycosylation sites (N184-G185-A186T, K368N-S369-S370) (Figure 1 and Supplement Figure S1). The predicted three-dimensional (3D) structure of structurally uncharacterized proteins can help us verify the viability of these mutations. We obtained the 3D structure of DSPAα1 from AlphaFold and exegesised the functional domains and sites by PyMOL (Figure 2, Figure 3 and Figure 4). We predicted the impact of these sites on the structure and function of DSPAα1 using site distance information of the 3D structure. 

DSPAα1 is highly fibrin selective, which is positively correlated with the F, the K, the P domains, and lacking plasmin-sensitive activation sites. Fibrin selectivity of DSPAα1 correlates with K43 and K65 in the F region [12]. K43 and K65 form a stable cross-like structure by interacting with a series of amino acids (D44, R59, R66, V67, E68) and participate in the recognition of fibrin (Figure 2C). The ligand-binding sites (W152, Y162, W190, Y192, and S201) in the K1 domain of DSPAα1is homologous to the FN II structure in fibronectin [46]. The ligand-binding sites in the FN type II structure primarily conciliates the recognition and binding of substrates, such as fibrin. The ligand-binding sites are connected by sorts of spatial forces to form a semi-open spherical structure to recognize fibrin (Figure 2D). DSPAα1 exerts its fibrinolytic activity by relying on the active pocket structure in its P domain. The structure consists of three parts: the active loop in I234-F244, active-associated sites (H272-D321-S428), and the salt bridge formed by K379-D427 (Figure 2E). The active loop mainly relies on the interaction between the four benzene rings and the connection between amino acid residues to keep stability. The three active sites are connected by hydrogen bonds. The salt bridge connects H272-D321-S428, and the active loop maintains the stability of the pocket structure (Figure 2E). The active pocket can specifically recognize the fibrin-plasminogen conjugate and use the pocket structure to encapsulate the conjugate and exhibit extremely high plasminogen activation activity.

From the predicted 3D structure, all mutation sites are located close to the active region or sites of DSPAα1. N153Q-S154-S155 and N184-G185-A186T are located close to the fibrin-selective sites and the ligand-binding sites, while K368N-S369-S370 and N398Q-K399-T400 are positioned close to the active pocket. As can be seen from Figure 3A–D, none of the mutation sites changed their original polar neighbor distances. Therefore, when these sites were mutated, we believed that the functional or structural changes brought about by changes in the amino acid properties were not considered.

To account for the accuracy and variability of protein structure, we define two amino acid residues as residues that form contact and the distance between them is less than 10 Å [47]. We used PyMOL to calculate the change of spatial distances and adjacent forces before and after mutation. N153Q-S154-S155 is located in the K1 region of DSPAα1 but is structurally close to the ligand-binding sites (9.2 Å). While N153 mutated to Q153, the spatial distance to the ligand-binding sites grow to 11.2 Å (Figure 4A). Changes in spatial distances indicate changes in steric hindrance between sites and domains, which have an impact on structure and function. N184-G185-A186T is very similar to the N185 site on DSPAα2 in terms of sequence and structure. Considering that DSPAα1 and DSPAα2 are highly homologous, we decided to generate an A186T mutant to make N184 acquire N-glycan chains and verify its effect on DSPAα1 activity. The spatial distance between N184-G185-A186T and the ligand-binding sites had unchanged (9.5 Å, Figure 4B). The N-glycan chains on N184 may participate in the interaction of ligand-binding sites, thus affecting structure and function. K368N-S369-S370 is highly homologous in sequence and structure to N322 on u-PA. The N-glycan chains on N322 are positively correlated with u-PA activity [48]. Therefore, we hypothesized that when K368 was mutated to N368, the N-glycan chains attached to K368N-S369-S370 may also affect DSPAα1 activity to a certain extent. According to the prediction (Figure 4C), K368N-S369-S370 is 17 Å far from the active pocket before mutation. The spatial distance after the mutation is reduced to 12 Å which was highly likely to produce steric hindrance with the presence of N-glycan chains. As such, the N-glycan chains attached to K368N-S369-S370 may affect the activity and specificity of DSPAα1 by affecting the stability of the active pocket. N398Q-K399-T400 is located in the P region of DSPAα1 and is also close to the active pocket (9.6 Å). However, when the sites mutated, the spatial distances between the active pocket and N398Q-K399-T400 grow up to 17 Å (Figure 4D). Therefore, the structural stability and activity of DSPAα1 may be reduced after the mutation. In addition, after these sites mutated, the adjacent forces also changed, indicating a change in steric hindrance.

In summary, we identified four potential N-glycosylation sites on DSPAα1 (N153Q-S154-S155, N184-G185-A186T, K368N-S369-S370, and N398Q-K399-T400). Further, the feasibility of these sites was analyzed at the structural level. Detailed information about the sites is stated in Table 2.

### 3.2. Expression, Purification, and SDS-PAGE Analysis of DSPAα1 and Mutants

All clones were detected by PCR and genomic RT-PCR to confirm the correct expression of the target protein (Supplement Figure S2). For *P. pastoris*, the linearization of *Sal* I usually generates multicopy clones, so we compared the relative copy numbers of rDSPAα1 and its N-glycosylation mutants. It was found by genomic qPCR that the relative copy numbers of rDSPAα1 and each mutant were at the same level (Supplement Figure S3). The concentration of the purified samples unified activity by the fibrin plate method, and the enzyme activity of each sample was obtained using the t-PA standard as the standard curve. The effect of N-glycosylation on protein yields is often reported, but its theory has not been determined. We found that rDSPAα1 expression was the highest for all samples (Supplement Table S2). Expression of QNGlyα1-1 was 33% lower than rDSPAα1 and QNGlyα1 and QNGlyα1-2 expression was 90% lower than rDSPAα1 (Supplement Table S2). These results suggest that deletion of N-glycan chains may result in decreased expression of rDSPAα1 in *P. pastoris*, while the impact of N398Q-K399-T400 on rDSPAα1 expression in the same expression system is much greater than that of N153Q-S154-S155. Expression of ANGlyα1 + 2 was 88% of rDSPAα1, and ANGlyα1 + U was 82% of rDSPAα1 (Supplement Table S2). Considering that both the Bradford method and fibrin plate method have certain errors, we believe that the expression levels of ANGlyα1 + 2 and ANGlyα1 + U should be consistent with rDSPAα1. So, we suggest that the addition of N-glycan chains on N184-G185-A186T and K368N-S369-S370 may not cause any change in expression of DSPAα1 in *P. pastoris*.

**Figure 5:**
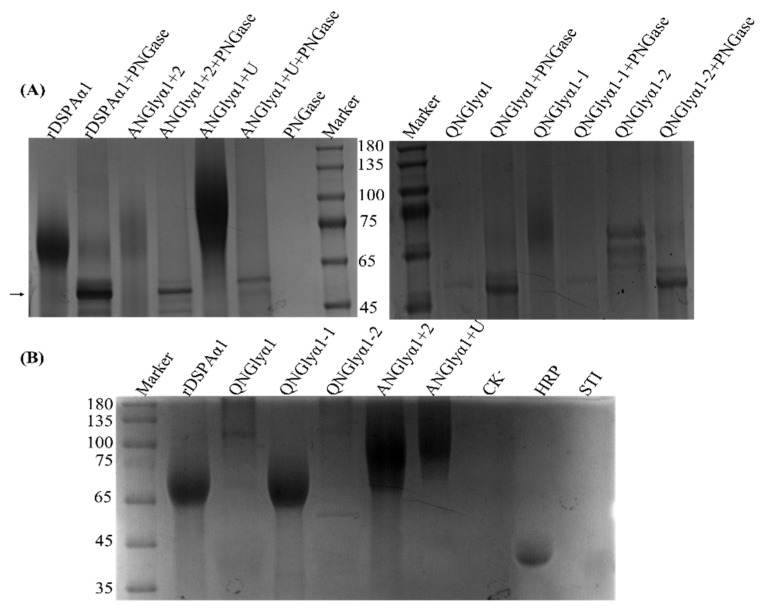
PNGase digestion and Carbohydrate staining of purified recombinant enzymes. (**A**) Coomassie blue staining. (**B**) Carbohydrate staining. Lanes: CK^−^, the cultivated supernatant of untransformed *P. pastoris*; Marker, molecular mass markers purchased from Zoman Bio; HRP, horseradish peroxidase (containing 16% N-glycoprotein, and its molecular weight is about 40 kDa); STI, soybean trypsin inhibitor (unglycosylated and therefore cannot be stained). The arrow showed the bands of wild and all mutants with PNGase F treatment, their molecular weight was consistent with that of QNGlyα1 without PNGase F treatment.

Protein samples were further characterized by SDS-PAGE. Glycosylation is known to change the molecular weight of proteins [49]. Furthermore, N-glycosylation can also cause smearing of the SDS-PAGE band because of the hydration effects. SDS-PAGE analysis showed that the size of QNGlyα1 with all N-glycosylation sites removed was approximately 52 kDa with no smearing (Figure 5A). QNGlyα1-2, which only retains N153Q-S154-S155, was 3 kDa larger than QNGlyα1. Moreover, we found that rDSPAα1 is 68 kDa, while QNGlyα1-1 which only retains N398Q-K399-T400 is 65 kDa. The N-glycan chains on N398Q-K399-T400 were more complex than those on N153Q-S154-S155 because the QNGlyα1-2 was 10 kDa larger than QNGlyα1-1. Compared with the original protein, ANGlyα1 + U were about 75 kDa, while ANGlyα1 + 2 was slightly smaller than ANGlyα1 + U. Following deglycosylation of N-glycan chains with PNGase F, the molecular weight of all samples was reduced to 52 kDa, which is consistent with the size of QNGlyα1 (Figure 5A). In addition, we also performed carbohydrate staining for auxiliary identification to prove that all N-glycosylation sites on the mutants have been N-glycosylated. Most samples can be properly stained with the carbohydrate staining kit (Figure 5B). A blurry stained band also appeared in the lanes QNGlyα1 which was without N-glycosylation. According to NetOGlyc-4.0 (https://services.healthtech.dtu.dk/services/NetOGlyc-4.0/, accessed on 10 September 2022), there are several potential O-glycosylation sites in DSPAα1. Therefore, we believe that this blurry band may be due to O-glycosylation.

### 3.3. Effect of N-Glycosylation on Thermostability and Acid-Base Tolerance

**Figure 6:**
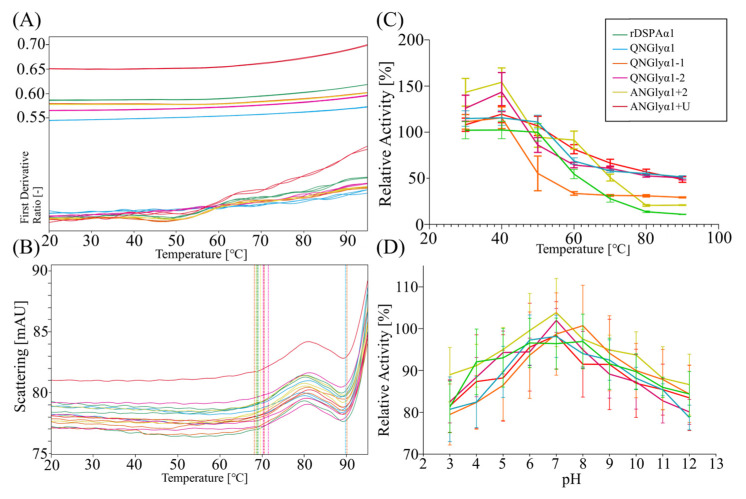
Thermostability and acid-base tolerance of rDSPAα1 and its mutants. Different colors represent the different protein samples: green represents rDSPAα1, blue represents QNGlyα1, orange represents QNGlyα1-1, purple represents QNGlyα1-2, yellow represents ANGlyα1 + 2, and red represents ANGlyα1 + U. (**A**) The F350/F330 ratio curve versus temperature during thermal denaturation. (**B**) The aggregation of protein samples during thermal denaturation. (**C**) In comparison to the activity of DSPAα1 and its mutants at different temperatures, the activity of different mutants at 4 °C was defined as 100% and the activity at other temperatures was compared with the activity at 4 °C to obtain their relative activity. The data were presented as the means ± SD (*n* = 3). (**D**) Comparison of the activity of DSPAα1 and its mutants at different pH, the activities of the different mutants at pH 7.0 were defined as 100%, and the activities at other pHs was compared with the activities at pH 7.0 to obtain their relative activities. The data were presented as the means ± SD (*n* = 3).

In general, N-glycosylation enhances protein stability [50]. We need to confirm whether N-glycosylation mutations alter its thermostability and acid-base tolerance. As a drug protein, DSPAα1 must be active and stable in normal blood which has a pH of 7.4 and a temperature of approximately 36.9–37.9 °C. We measured the samples by nanoDSF. The F350 nm/F330 nm ratio of the samples all started at 90 °C (Figure 6A). The scattered signal trend is consistent across these samples. The scattered signal of all samples was grown at around 69 °C, indicating that all six proteins gathered at 69 °C. They all increased sharply around 90 °C, indicating significant protein aggregation from 90 °C (Figure 6B). We suggested that these samples unfolded began at 90 °C. Using S-2765™ to monitor changes in protein activity at different temperatures and pH. We showed that the activity vs. temperature curves for the six protein samples were highly consistent (Figure 6C). All protein samples showed increased activity at 30–40 °C. The activity curves versus the pH of the six protein samples were highly consistent too (Figure 6D). All protein samples showed high catalytic activity towards S-2765 under pH 6.0–7.0. All protein samples showed decreased activity in acidic (pH 3.0–5.0) or alkaline (pH 9.0–12.0) environments. 

In sum, both rDSPAα1 and its N-glycan mutants exhibited consistent environmental tolerance (optimal pH, temperature, and thermal stability). Therefore, N-glycosylation mutations do not alter the physicochemical properties of DSPAα1.

### 3.4. Effect of N-Glycosylation on Fibrinolytic Activity and Fibrin Selectivity of DSPAα1 and Its Mutants In Vitro

**Figure 7:**
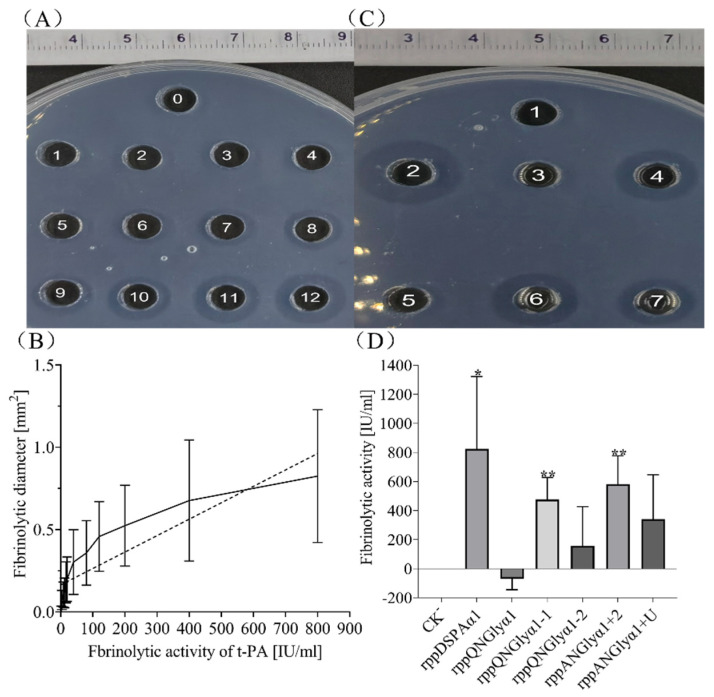
The fibrinolytic activity of rDSPAα1 and its mutants. (**A**) The dissolving area of gradient enzymatic t-PA in fibrin plates, wells 0–12, represents the gradient enzyme activity of t-PA used in the experiment (0, 2, 4, 8, 12, 16, 20, 40, 80, 120, 200, 400, 800 IU/mL). (**B**) The standard curve of fibrinolytic diameter versus fibrinolytic activity. (**C**) The dissolved area of DSPAα1 and its mutants in fibrin plates, wells 1–7: CK^−^, rDSPAα1, QNGlyα1, QNGlyα1-1, QNGlyα1-2, ANGlyα1 + 2, ANGlyα1 + U. (**D**) The relative enzymatic activities of DSPAα1 and its mutants. The significant differences between means were evaluated by *t*-test and using rDSPAα1 as a control. * = 0.01 ≤ *p* ≤ 0.05, ** = *p* ≤ 0.01.

We confirmed the relative fibrinolytic activity of each protein sample by the fibrin plate method (Figure 7). We established a standard curve between thrombolytic area and enzyme activity using the standard t-PA (Figure 7A,B). The fibrinolytic activity of rDSPAα1 was the highest among all samples, followed by ANGlyα1 + 2 and QNGlyα1-1, with QNGlyα1 activity being almost nonexistent (Figure 7C). Deletion of N-glycan chains on N153Q-S154-S155 resulted in decreased activity of DSPAα1, and the activity of QNGlyα1-1 was 57% of rDSPAα1 (Figure 7D). The fibrinolytic activity of QNGlyα1-2 is only 19% of rDSPAα1, which is much lower than QNGlyα1-1 (Figure 7D). The QNGlyα1 with deglycosylation mutations at the two sites lost almost all of its fibrinolytic activity (1% of rDSPAα1) compared to the wild-type protein (Figure 7D).

The introduction of N-glycan chains on N184-G185-A186T and K368N-S369-S370 did not increase the activity as expected. ANGlyα1 + 2 and ANGlyα1 + U activity was only 70% and 40% of rDSPAα1, respectively (Figure 7D). According to Figure 4B, the N-glycan chains on N184-G185-A186T might interfere with the structural stabilization of the ligand-binding sites involved in the recognition of fibrin, thereby leading to the reduction of fibrinolytic activity. The N-glycan chains on K368N-S369-S370, which interfered with the structure of the active pocket, significantly reduced the activity. According to Figure 4C, K368N-S369-S370 is spatially located close to the active pocket which is an important structural feature of DSPAα1 to exert fibrinolytic activity. When N-glycan chains attached to K368N-S369-S370, the chain was likely to be inserted into the active pocket and destroy the structure.

To verify the effect of these N-glycosylation sites mutation on the fibrin selectivity of DSPAα1, the S-2765 substrate method was applied to compare differences in the activity of each protein sample under different cofactors (Table 3). We found that rDSPAα1 showed the highest fibrin selectivity and fibrinolytic activity in all samples. The *K*cat/*K*m of fibrin as a cofactor was 169 times higher than that without a cofactor and 21 times higher than fibrinogen as a cofactor (Table 3). QNGlyα1 showed the lowest fibrinolytic activity and fibrin selectivity among all samples. The *K*cat/*K*m of fibrin as a cofactor was 3.5 times that without a cofactor and only 0.7 times that with fibrinogen as a cofactor (Table 3). Compared with rDSPAα1, the *K*cat/*K*m of QNGlyα1-1 in the absence of cofactors was comparable to that of rDSPAα1, but when fibrin was used as a cofactor, its *K*cat/*K*m was only 5.6% of rDSPAα1 (Table 3), showing extremely low fibrin selectivity. The *K*cat/*K*m of ANGlyα1 + 2 was 79% of rDSPAα1 when fibrin was used as a cofactor, which was consistent with the results obtained by the fibrin plate method (Table 3).

Considering that the fibrin selectivity is associated with an unspecified feature of the P region, we compared the effect of N-glycan chains on K368N-S369-S370 and N398Q-K399-T400 on DSPAα1. Compared with rDSPAα1, the fibrin selectivity of QNGlyα1-2 decreased significantly to only 6% of rDSPAα1. However, compared with QNGlyα1-1, QNGlyα1-2 showed higher fibrin selectivity, and the ratio of QNGlyα1-2 was higher too (Table 3). Fbn/Fbg was approximately 7.7 times higher than that of QNGlyα1-1 (Table 3), which further indicated that the fibrin selectivity of DSPAα1 is related to various structural features of DSPAα1, and the N-glycan chains on N153Q-S154-S155 had a more significant effect than N398Q-K399-T400. However, the fibrinolytic activity and fibrin selectivity of ANGlyα1 + U were at deficient levels consistent with QNGlyα1. We suspected this might be related to the structure of the active pocket affecting DSPAα1. The fibrinolytic activity of ANGlyα1 + U is at a very low level (Figure 7, Table 3). When the fibrinolytic activity of DSPAα1 was extremely low, its fibrin selectivity could not be exerted. Therefore, the fibrin selectivity of ANGlyα1 + U could not be detected and thus was at deficient levels consistent with that of QNGlyα1-1.

In conclusion, we verified that N-glycosylation has a significant influence on the activity of DSPAα1 by either the fibrin plate method or the S-2765 substrate method. N153Q-S154-S155 is highly correlated with the fibrin selectivity of DSPAα1 but has little effect on the fibrinolytic activity, while N398Q-K399-T400 has a significant impact on the fibrin selectivity and fibrinolytic activity. The N-glycan chains on N184-G185-A186T did not increase the fibrinolytic activity and fibrin selectivity of DSPAα1 as expected and even caused a slight decrease in activity. The N-glycan chains on K368N-S369-S370 cause loss of fibrin selectivity and fibrinolytic activity in DSPAα1.

### 3.5. Effects of N-Glycosylation Mutations on Thrombolytic Effects of DSPAα1 and Its Mutants in Rats

According to the results of the activity experiments in vitro, we screened out ANGlyα1 + 2 and QNGlyα1 as the two mutants with the highest or the lowest activity, respectively, for experiments in vivo in SD rats. Subsequently, rDSPAα1 was used as a positive control to participate in the experiment. At the same time, rt-PA was introduced as a reference. Mg and Ck groups served as negative controls.

The recanalization rate is the most intuitive means to judge the effect of thrombolysis [51]. The unmetabolized blood vessel at the distal end was ligated, and the wire knot near the heart was cut off at the end of the thrombus. Whether the thrombus was successfully recanalized was judged by whether there was blood flow out. Statistical analysis showed that the recanalization rate of rDSPAα1 was the highest (4/8) compared to 3/8 for ANGlyα1 + 2 and rt-PA, and 0/8 for QNGlyα1 (Supplement Table S3). The recanalization results were consistent with those obtained from the in vitro experiments. The simultaneous loss of N153Q-S154-S155 and N398Q-K399-T400 resulted in an almost inactive DSPAα1.

**Figure 8:**
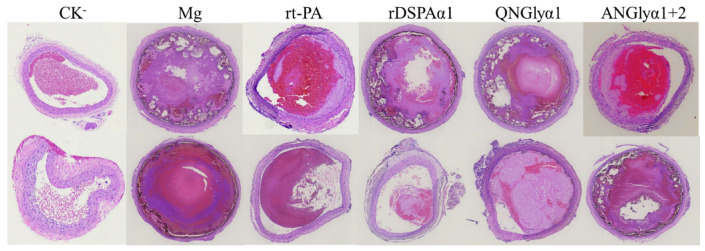
H & E staining sections of rat thrombus.

The thrombolytic effect of different thrombolytic proteins can not be well judged by relying on the recanalization rate. Pathological sections are often observed and adapted. Therefore, staining on the sections of the thrombus and developing a scoring standard can improve the visualization of thrombolysis effects (Figure 8, Table 4) [52]. In this experiment, thrombus sections were scored through six indexes, and a total score was finally obtained to evaluate the thrombolytic effect of these protein samples. Regarding Figure 8 and Table 4, the rDSPAα1 group had the lowest score while the QNGlyα1 group had the highest score even approaching the Mg group. The ANGlyα1 + 2 and rt-PA groups had almost the same score. According to the thrombus fissure scores alone, the rDSPAα1 group still scored the lowest, followed by the rt-PA group. However, the score of the ANGlyα1 + 2 group was similar to the QNGlyα1 group, both of which were close to the Mg group (Table 4). In summary, rDSPAα1 has the highest thrombolytic efficiency, and the N-glycosylation mutations lead to reduced thrombolytic efficiency.

**Figure 9:**
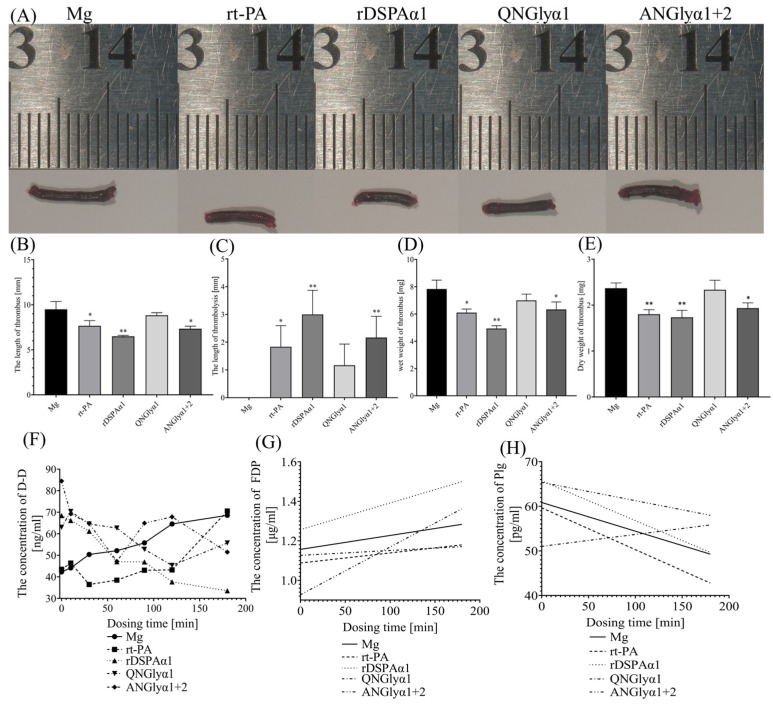
The comparison of three indicators of in vitro thrombosis detection and concentration of thrombopathological factors of rDSPAα1 and its mutants in rats. (**A**) Length of thrombus in rats, all samples are measured to the nearest mm; (**B**) Length of thrombus; (**C**) Length of thrombolysis; (**D**) The wet weight of thrombus; (**E**) Dry weight of the thrombus; (**F**) Curve of D-D concentration versus time; (**G**) Standard curve of FDP concentration versus time. (**H**) Standard curve of Plg concentration versus time. The significant differences between means were evaluated by *t*-test and using the Mg group as a control. * = 0.01 ≤ *p* ≤ 0.05, ** = *p* ≤ 0.01.

Determining the length, dry weight, and wet weight of thrombus can help to predict thrombus severity in the body and promote research on thrombus [53]. The differences in activity between different proteins were compared by analyzing the length (Figure 9B,C), dry weight (Figure 9D), and wet weight (Figure 9E) of thrombus in rats treated with different protein samples. The dry weight, wet weight, and length of the arterial thrombus in group rDSPAα1, rt-PA, and ANGlyα1 + 2 were significantly different from those in the Mg group (*p* < 0.05), but no statistically significant differences were observed in the QNGlyα1 group (*p* > 0.05) (Figure 9B–E). Therefore, we introduced thrombolytic lengths for comparison. The average thrombus length of the Mg group was used as the original length for each group and the average thrombus length for each group was subtracted to obtain the thrombolytic length (Figure 9C). The rDSPAα1 activity was the highest, followed by rt-PA and ANGlyα1, with QNGlyα1 having the lowest activity. The differences in the wet weight of the thrombus between groups were not as obvious as the difference in the dry weight of the thrombus (Figure 9D,E). The dry and wet weights of rDSPAα1 were both the lowest. The dry and wet weights of rt-PA and ANGlyα1 + 2 were larger than those of rDSPAα1, but the differences were minor. The dry and wet weights of QNGlyα1 were not significantly different from those of the Mg group, indicating a poor thrombolytic effect of QNGlyα1.

The trends of concentration of thrombopathological factors over time can also reflect the efficiency of thrombolysis. In this experiment, three factors that are highly correlated with thrombolysis (D-dimer, FDP and Plg) were selected.

D-dimer can reflect the change in the size of the thrombus so that it can be used for curative effect observation of thrombolytic therapy. If D-dimer continues to be high or repeated increases during the treatment period, the treatment is ineffective or incomplete [54]. The QNGlyα1 group continued to increase during the treatment period as the Mg group, indicating that the thrombolysis efficiency was extremely low (Figure 9F). The rt-PA group experienced multiple repetitions during the treatment period and increased sharply 3 h after administration, indicating that the half-life of rt-PA is shorter, and the recurrence rate of thrombus is high (Figure 9F). The D-dimer concentration in the rDSPAα1 group decreased during the treatment period, indicating that the thrombolytic effect was excellent (Figure 9F). It is worth noting that, although the ANGlyα1 + 2 group also experienced repeated recurrences during the treatment period, finally the concentration of D-dimer was controlled at a low level after 3 h of administration (Figure 9F). This result shows that N-glycan chains attached to N184-G185-A186T did not affect the stability of DSPAα1, which has also been proved before (Figure 6). The reduction of fibrin selectivity results in an unstable thrombolytic process of ANGlyα1 + 2, resulting in repeated increases in the concentration of D-dimer.

FDP mainly reflects fibrinolytic function. FDP levels should be at normal levels during treatment. If there is a sharp increase or little change during treatment, the thrombolysis is less effective and side effects may occur [55]. Taking the Mg group as a control, the slope of the rDSPAα1 group was slightly larger than the Mg group, indicating that rDSPAα1 had an excellent thrombolytic effect (Figure 9G). The slope of the rt-PA group was almost the same as the Mg group, suggesting that the thrombolytic result was general (Figure 9G). The slope of group QNGlyα1 is 0.092 smaller than the Mg group, as QNGlyα1 almost lost all thrombolytic activity (Figure 9G). The slope of group ANGlyα1 + 2 was much larger than that of group Mg and rDSPAα1, indicating that although ANGlyα1 + 2 had a thrombolytic effect, side effects (e.g., hyper-fibrinolysis, bleeding) may have occurred because of the low fibrin selectivity (Figure 9G).

Plasminogen is the main substrate of rt-PA and DSPAα1. During treatment, the concentration of plasminogen can intuitively reflect the plasminogen activation efficiency of the protein [56]. We still used the slope of the Mg group as a control. The slope of the rDSPAα1 group and the rt-PA group were almost the same but slightly smaller than those of the Mg group, indicating that the activation efficiency of plasminogen was higher (Figure 9H). The slope of the ANGlyα1 + 2 group was slightly larger than that of the Mg group, indicating that the plasminogen activation efficiency was not as good as rDSPAα1 and rt-PA. The slope of the QNGlyα1 group was the only positive number among all groups, with almost no plasminogen activation efficiency (Figure 9H).

In summary, the simultaneous loss of N-glycan chains on N153Q-S154-S155 and N398Q-K399-T400 not only causes DSPAα1 to lose its plasminogen activation activity and thrombolytic effect but also significantly reduces the fibrin selectivity of DSPAα1. This affects DSPAα1 safety in vivo. The addition of N-glycan chains on N184-G185-A186T leads to slightly reduced plasminogen activation activity in DSPAα1, but the thrombolytic effect should still be maintained at the same level as rt-PA. The main effect of N-glycan chains is to decrease the fibrin selectivity of DSPAα1, causing hyper-fibrinolysis after dosing.

### 3.6. Effects of N-Glycosylation Mutations on the Thrombolysis Safety of DSPAα1 and Its Mutants in Rats

DSPAα1 is well known for its specific thrombolytic activity, but N-glycosylation mutations lead to a decrease in its fibrin selectivity, therefore, its thrombolysis safety in rats must be verified. DSPAα1 does not cause a decrease in platelet numbers after use and, therefore, does not disrupt coagulation. From these experimental data, rDSPAα1, QNGlyα1, and ANGlyα1 + 2 all showed a significant reduction in platelet count after 15 min of medication, but after 1 h of medication, the platelet count returned to similar levels as the Mg group, while the rt-PA group was treated resulting in a significant decrease in platelet count after 15 min and 1 h after administration and did not return to the level of Mg group (Table 5). This shows that rDSPAα1 has superior drug safety. Therefore, whether the N-glycosylation mutations occurred was unlikely to cause a reduction in platelet numbers.

**Figure 10:**
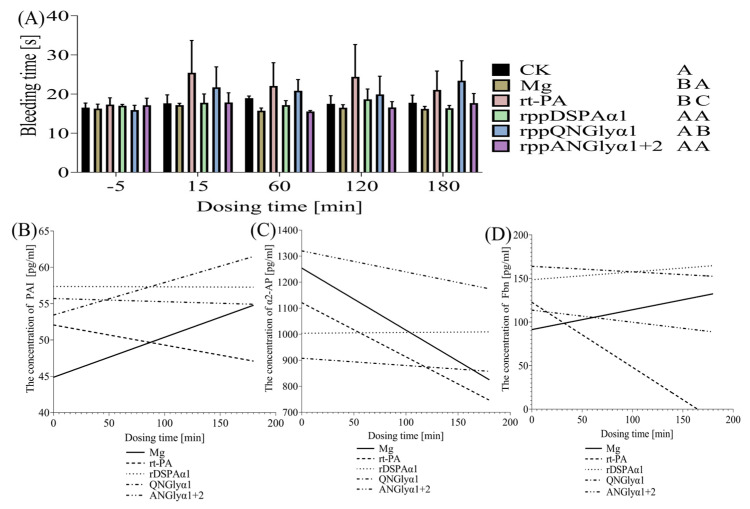
Comparison of bleeding time and concentration of coagulation-related factors with a dosing time of rDSPAα1 and its mutants in rats. (**A**) Monitoring the bleeding time in rat hindlimb veins. The significant differences between means were evaluated by *t*-test and using the Ck and Mg group as a control separately. AB = 0.01 ≤ *p* ≤ 0.05, AC = *p* ≤ 0.01. (**B**) Standard curve of PAI concentration versus time. (**C**) Standard curve of α2-AP concentration versus time. (**D**) Standard curve of Fbn concentration versus time.

Bleeding time can well reflect whether the balance between the fibrinolytic and coagulation systems is safe [57]. In general, the addition of thrombolytic drugs causes an imbalance between the fibrinolytic and coagulation systems in vivo, but this effect does not last long. Hyper-fibrinolysis can occur if the thrombolytic drug causes the system to become unbalanced, leading to irregular bleeding. The effect of protein samples in rats was determined by monitoring the bleeding time at multiple time points before and after administration (Figure 10A). Due to the presence of a thrombus, the bleeding time in the Mg group was shorter than in all other groups at each time point. The rDSPAα1 and ANGlyα1 + 2 groups maintained a relatively stable bleeding time during the medication period, and the time was similar to that of the Mg group (Figure 10A). The bleeding time in the rt-PA group was significantly higher than that of the Mg group 15–120 min after administration, and the bleeding time at 180 min returned to the same level as group Mg, which may be related to its degradation in vivo (Figure 10A). The bleeding time in the QNGlyα1 group was significantly longer than that in the Mg group at 15–180 min after administration (Figure 10A). rDSPAα1 had the lowest bleeding rate, and the removal of the rude N-glycosylation sites resulted in an increased bleeding rate.

PAI, α2-AP, and Fbn are essential indicators to investigate whether the fibrinolytic and coagulation systems are unbalanced in rats and are often used to monitor the safety of thrombolytic drugs in clinical practice. PAI-1, one form of PAI, can bind to plasminogen activators such as u-PA and t-PA in a 1:1 ratio to activate plasminogen [58]. DSPAα1 does not combine with PAI-1, so it does not consume PAI-1 excessively and can therefore safely maintain the stability of the coagulation system in the body. The PAI concentration of the rDSPAα1 and ANGlyα1 + 2 group maintained a relatively stable level during the monitoring period, while the PAI concentration of the rt-PA group continued to decrease during the monitoring period, and the PAI concentration of the QNGlyα1 group was consistent with the Mg group and continued to increase during the monitoring period (Figure 10B). Further, α2-AP is synthesized by the liver and can bind to plasmin (PL) 1:1 to inhibit PL activity [59]. Activation of plasmin from plasminogen by DSPAα1 promotes further binding to plasmin, thus hindering inhibition of PL by α2-AP. Therefore, this will not consume much α2-AP. Both rDSPAα1 and QNGlyα1 maintained a relatively stable α2-AP concentration during the monitoring period, while the α2-AP concentration in the ANGlyα1 + 2 group decreased during the monitoring period. However, the proportion of the decrease was much lower than that in the rt-PA and the Mg group (Figure 10C). Fbn is the main component involved in the coagulation system in vivo, therefore, the concentration of Fbn is related to the stability of the coagulation system [60]. DSPAα1 has a very high fibrin selectivity and shows deficient fibrinolytic activity in the absence of cofactors or fibrinogen as a cofactor, meaning, it will not consume too much fibrinogen. The Fbn concentration of the rDSPAα1, QNGlyα1, and ANGlyα1 + 2 groups did not change significantly during the monitoring period. Moreover, the Fbn concentration of the Mg group showed a slow upward trend during the monitoring period, while the rt-PA group showed a significant decrease (Figure 10D).

Above all, rDSPAα1 expressed by *P. pastoris* does not cause adverse reactions in rats. The N-glycan chains on N153Q-S154-S155 and N398Q-K399-T400 can affect the safety of DSPAα1 in rats because prolonged bleeding time increases the risk of irregular bleeding. The introduction of N-glycan chains to N184-G185-A186T did not increase the safety risk of DSPAα1 although its fibrin selectivity has been reduced.

## 4. Discussions

Reviewing the research data in recent years, the research progress of thrombolytic drugs has been relatively slow, the discovery rate of new drugs is extremely low, and artificially modified drugs are often not ideal. DSPAα1 has been investigated as a new thrombolytic drug since its discovery. Its superior fibrin selectivity and long half-life are not available in the existing third-generation thrombolytic drugs. However, its expression level, purification process, and cost, the immunogenicity of N-glycan chains, and side effects have seriously hindered its clinical application.

The rational design of N-glycosylation sites may provide a new research idea for DSPAα1 mutants on how to maintain or improve their activity. In this study, by means of homology alignment and predictive modeling, a total of 5 N-glycosylation mutants were constructed, which proved the effectiveness of N-glycosylation on the activity of DSPAα1. The active region of protein which is highly correlated with protein activity or function is often composed of a variety of complex secondary structures. At positions involved in α-helical or β-sheet secondary structure, N-glycosylation tends to destabilize the protein, presumably because the bulky N-glycan disrupts important native state interactions [61]. Through structure prediction, we found that N184-G185-A186T and K368N-S369-S370 are highly close to the active region of DSPAα1. So, we speculated that the introduction of N-glycans at these two N-glycosylation sites might lower the activity-related structure of DSPAα1. Related wet experiments also confirm this prediction (Figure 7D, Table 3). Compared with t-PA, the fibrin selectivity of DSPAα1 is a main advantage as a thrombolytic drug [13]. DSPAα1 specifically activates plasminogen which is bound to fibrin as plasmin to promote thrombolysis [15]. The fibrin selectivity of DSPAα1 results in its little activity in the absence of fibrin [16]. However, inappropriate N-glycosylation mutations can lead to reduced fibrin selectivity. The mutants with reduced fibrin selectivity (QNGlyα1, QNGlyα1-1, ANGlyα1 + 2) showed different degrees of reduced fibrinolytic activity (Figure 7C,D). However, the reduced fibrin selectivity would result in some fibrinolytic activity of DSPAα1 in the absence of fibrin. According to in vitro data, the fibrin selectivity of both QNGlyα1 and ANGlyα1 + 2 decreased. QNGlyα1 and ANGlyα1 + 2 presented higher activity than rDSPAα1 in the absence of cofactors in vitro (Table 3). In vivo, QNGlyα1 and ANGlyα1 + 2 were worse than rDSPAα1 in all thrombolytic indexes (Figure 9), while the side effects were more serious than rDSPAα1, especially QNGlyα1 (Figure 10). We have known that mutations at these sites lead to a decrease in fibrin selectivity through rational predictions (Figure 4). The using of rational design can help researchers avoid unnecessary wet experiments. In the follow-up study of DSPAα1, crystal structures or homology modeling studies should help to further analyze the spatial relationship and interaction force between the N-glycan chains on DSPAα1 and its active sites [62].

Furthermore, the effects of different N-glycosylation sites on the activity of DSPAα1 are inconsistent. In vitro, the N-glycan chains on N153Q-S154-S155 and N398Q-K399-T400 were highly correlated with the fibrinolytic activity of DSPAα1. The fibrinolytic activity of QNGlyα1-2 is much lower than QNGlyα1-1 (Figure 7D, Table 3). So, we suggested that the deletion of N-glycan chains on N398Q-K399-T400 had a more significant effect on DSPAα1 activity compared to N153Q-S154-S155. The addition of N-glycosylation sites at N184-G185-A186T and K368N-S369-S370 also led to a certain degree of reduction in both fibrinolytic activity and fibrin selectivity. N-glycan chains on N184-G185-A186T have a great effect on fibrin selectivity while the N-glycan chains on K368N-S369-S370 have great effects on fibrinolytic activity and fibrin selectivity (Figure 7D, Table 3). In vivo experiments have also made corresponding findings. In vivo, the fibrinolytic activity of QNGlyα1 is almost lost while ANGlyα1 + 2 is slightly lower than that of DSPAα1 (Figure 9). They all increase the hemolysis risk while the side effects of QNGlyα1 were more obvious (Figure 10). These results also suggest that there may be other regulatory mechanisms in the body.

N-glycan chains are extremely important for the activity of DSPAα1. The deletion or change of the N-glycosylation site will lead to significant changes in the fibrin selectivity and fibrinolytic activity of DSPAα1. In addition to changing the N-glycosylation site, the humanized N-glycosylation modification of DSPAα1 may also be a method to reduce immunogenicity, which can be achieved in the *P. pastoris* with human N-glycosylation modified [63]. Moreover, investigating the preparation of HSA fusions and pegylation on DSPAα1 was another way to avoid the immune response. HSA is the most abundant soluble protein in human plasma. Using HSA as a carrier for fusion expression with a drug protein can effectively prolong the half-life of drugs in plasma and avoid immune responses [64]. We believe that modifying the existing characteristics of DSPAα1 would be a more time- and cost-saving solution than developing and researching new drugs.

## Figures and Tables

**Table 1:** Sampling time point.

Administration	Before	After					
Sampling time (min)	−5.000	10.000	30.000	60.000	90.000	120.000	180.000
blood volume (μL)	300.000	300.000	300.000	300.000	300.000	300.000	300.000
TBT (min)	−5.000	10.000	30.000	60.000	90.000	120.000	180.000

**Table 2:** Detailed information about N-glycosylation sites of DSPA α1 and its mutants.

Enzyme	Motifs	Modification	Mutation Sites	N-Glycosylation Sites
rDSPAα1	N153-N184(A186)-K368-N398	/	/	N153-N398
QNGlyα1	Q153-N184(A186)-K368-Q398	Deletion	N153Q N398Q	/
QNGlyα1-1	Q153-N184(A186)-K368-N398	Deletion	N153Q	N398
QNGlyα1-2	N153-N184(A186)-K368-Q398	Deletion	N398Q	N153
ANGlyα1 + 2	N153-N184(T186)-K368-N398	Addition	A186T	N153-N184-N398
ANGlyα1 + U	N153-N184(A186)-N368-N398	Addition	K368N	N153-N368-N398

N-glycosylation sites are represented only by the site linked to the glycans. Mutation sites indicate sites that were directed mutated (A186 was mutated to T186 to make N184 a potential N-glycosylation site).

**Table 3:** Kinetic parameters of S-2765^TM^ hydrolysis by DSPAα1 and its mutants in the presence or absence of a fibrin(ogen) cofactor.

Enzyme	Cofactor	*K*_cat_ ×10^3^	*K*_m_ mM	*K*_cat_/*K*_m_ mM^−1^S^−1^	Stimulation Factor	Ratio Fbn/Fbg
rDSPAα1	None	0.094 ± 0.001	2.709 ± 0.155	34.830 ± 2.350	1.000	
	Fbg	0.097 ± 0.000	0.343 ± 0.015	282.240 ± 13.070	8.100	
	Fbn	0.259 ± 0.024	0.045 ± 0.010	5883.440 ± 983.570	168.900	20.800
QNGlyα1	None	0.084 ± 0.004	1.380 ± 0.257	62.240 ± 10.690	1.000	
	Fbg	0.118 ± 0.012	0.369 ± 0.077	328.740 ± 62.750	5.300	
	Fbn	0.165 ± 0.017	0.763 ± 0.085	216.420 ± 5.840	3.500	0.700
QNGlyα1-1	None	0.088 ± 0.001	2.258 ± 0.046	38.800 ± 0.780	1.000	
	Fbg	0.144 ± 0.019	0.493 ± 0.005	292.580 ± 1.780	7.500	
	Fbn	0.149 ± 0.001	0.448 ± 0.030	333.760 ± 20.680	8.600	1.100
QNGlyα1-2	None	0.138 ± 0.008	1.764 ± 0.111	78.240 ± 0.600	1.000	
	Fbg	0.126 ± 0.004	1.320 ± 0.033	95.230 ± 3.970	1.200	
	Fbn	0.124 ± 0.008	0.153 ± 0.016	812.780 ± 54.960	10.400	8.500
ANGlyα1 + 2	None	0.089 ± 0.001	3.688 ± 0.318	24.280 ± 1.990	1.000	
	Fbg	0.144 ± 0.003	0.577 ± 0.024	248.960 ± 6.330	10.300	
	Fbn	0.168 ± 0.027	0.052 ± 0.005	3257.040 ± 561.250	134.100	13.100
ANGlyα1 + U	None	0.082 ± 0.002	0.674 ± 0.067	122.880 ± 13.680	1.000	
	Fbg	0.096 ± 0.005	0.453 ± 0.129	221.760 ± 60.490	1.800	
	Fbn	0.097 ± 0.001	0.168 ± 0.028	590.130 ± 98.900	4.800	2.700

The kinetics of S-2765 hydrolysis was measured as described in “Materials and Methods”. The kinetic parameters were from a nonlinear regression analysis of the Michaelis–Menten plots, which depict the rate of pNA generation versus S-2765 concentration. The stimulators observed in the presence of fibrin (ogen) were shown in the second column from the right. It was calculated as the ratio of the bimolecular rate constant in the presence of fibrinogen or fibrin to the bimolecular rate constant in the absence of fibrinogen or fibrin. Fibrin selectivity in the right column is calculated as the ratio of activity (fibrin/fibrinogen). The rates for all samples differ significantly from each other.

**Table 4:** Scores of thrombopathological biopsy analysis.

Samples	P1	P2	P3	P4	P5	P6	Total Score
CK	0.000 ± 0.000	0.000 ± 0.000	0.000 ± 0.000	0.000 ± 0.000	0.000 ± 0.000	0.000 ± 0.000	0.000 ± 0.000
Mg	20.000 ± 0.000	20.000 ± 0.000	19.375 ± 0.354	19.375 ± 0.354	18.750 ± 0.463	16.875 ± 0.744	114.375 ± 0.835
rt-PA	15.833 ± 1.329	16.667 ± 1.211	12.500 ± 0.837	14.167 ± 0.753	10.833 ± 1.169	20.000 ± 0.000	90.000 ± 2.366
rDSPAα1	9.167 ± 1.169	14.167 ± 0.983	7.500 ± 0.548	7.500 ± 0.548	5.000 ± 0.000	18.333 ± 0.516	61.667 ± 1.966
QNGlyα1	15.833 ± 0.753	20.000 ± 0.000	13.333 ± 0.516	17.500 ± 0.547	20.000 ± 0.000	16.667 ± 0.816	103.333 ± 0.816
ANGlyα1 + 2	16.667 ± 0.816	20.000 ± 0.000	14.167 ± 0.753	15.000 ± 0.632	18.333 ± 0.516	8.333 ± 0.816	92.500 ± 1.049

Each item is worth 20 points, the total score is 120 points. The scoring rule of the thrombus component (P1, P2, P3) is that the higher the proportion, the higher the score. The scoring rule of thrombolytic components (P4, P5, P6) is that the lower the proportion, the higher the score. The total score of all samples has significant differences between each other.

**Table 5:** Platelet changes before and after dosing.

		PLT (10^9^/L)	MPV	PDW (%)	PCT	*p* Value
CK	No dosing	67.000 ± 7.211	9.400 ± 0.265	14.167 ± 0.462	0.062 ± 0.005	
	15 min after dosing	65.000 ± 1.732	9.633 ± 0.058	13.833 ± 0.306	0.062 ± 0.001	*p* > 0.05
	1 h after dosing	60.333 ± 4.163	9.700 ± 0.265	14.133 ± 0.643	0.062 ± 0.005	*p* > 0.05
Mg	No dosing	830.667 ± 37.072	7.700 ± 0.520	8.933 ± 0.666	0.640 ± 0.016	
	15 min after dosing	826.333 ± 50.143	7.600 ± 0.300	8.833 ± 0.551	0.628 ± 0.021	*p* > 0.05
	1 h after dosing	746.000 ± 42.673	7.600 ± 0.265	8.800 ± 0.520	0.567 ± 0.008	*p* < 0.005
rt-PA	No dosing	872.667 ± 105.945	6.833 ± 0.058	7.567 ± 0.231	0.594 ± 0.078	
	15 min after dosing	716.333 ± 106.931	7.133 ± 0.153	7.767 ± 0.404	0.512 ± 0.066	*p* > 0.05
	1 h after dosing	576.333 ± 42.595	6.933 ± 0.416	7.900 ± 0.721	0.401 ± 0.052	*p* < 0.005
rDSPAα1	No dosing	728.333 ± 73.173	7.367 ± 0.153	8.333 ± 0.231	0.537 ± 0.065	
	15 min after dosing	585.667 ± 71.654	7.133 ± 0.231	8.133 ± 0.451	0.417 ± 0.038	*p* < 0.0001
	1 h after dosing	724.333 ± 50.817	7.000 ± 0.100	7.867 ± 0.289	0.506 ± 0.028	*p* > 0.05
QNGlyα1	No dosing	860.667 ± 25.146	6.567 ± 0.115	7.300 ± 0.000	0.563 ± 0.028	
	15 min after dosing	772.667 ± 54.775	7.333 ± 0.289	8.167 ± 0.751	0.547 ± 0.067	*p* > 0.05
	1 h after dosing	498.000 ± 21.284	6.900 ± 0.265	7.867 ± 0.493	0.357 ± 0.031	*p* < 0.005
ANGlyα1 + 2	No dosing	768.000 ± 120.104	6.967 ± 0.503	7.900 ± 0.721	0.532 ± 0.048	
	15 min after dosing	572.333 ± 18.502	7.100 ± 0.436	7.900 ± 0.889	0.405 ± 0.014	*p* > 0.05
	1 h after dosing	712.667 ± 16.010	7.033 ± 0.643	7.900 ± 0.889	0.502 ± 0.055	*p* > 0.05

PLT: platelet count, MPV: mean of platelet volume, PDW: width of platelet distribution, and PCT: thrombocytosis. *p* values were calculated by comparing the post-dose data of each group with the pre-dose data.

## Data Availability

The data presented in this study are available on request from the corresponding author. The data are not publicly available due to non-disclosure agreement of the fund.

## Supplementary Materials

The following supporting information can be downloaded at: https://www.mdpi.com/article/10.3390/life13040985/s1, Supplement Figure S1: Alignment of amino acid sequences of DSPA α1, DSPA α2, t-PA, and u-PA; Supplement Figure S2: PCR and RT-PCR detection of rDSPAα1 and its mutations; Supplement Figure S3: Relative copies of rDSPAα1 and its mutants; Supplement Table S1: The oligonucleotide primers used in this study; Supplement Table S2: Protein yields of rDSPAα1 and its mutants after purification; Supplement Table S3: The recanalization rate of the thrombosis rat model.

## Abbreviations

**Abbreviations**	**Full Name**
Å	Ångström unit, the smallest unit representing the spatial distance between two sites or atoms
D-D	D-dimer
F	Fibrin
Fbn	Fibrinogen
FDP	Fibrin/Fibrinogen Degradation Products
HRP	Horseradish peroxidase containing 16% glycoprotein
*K*cat	Turnover number
*K*m	Michaelis constant
MPV	The mean of platelet volume
Plg	Plasminogen
PLT	The platelet count
PDW	The width of platelet distribution
PCT	Thrombocytosis
PAI	Plasminogen activator inhibitor
PNGase F	The most effective enzyme for removing N-linked glycans from glycoproteins
rt-PA	Alteplase
S-2765	A chromogenic substrate for determination of Factor Xa activity
STI	Soybean Trypsin Inhibitor, unglycosylated
SPF	Specific pathogen-free
t-PA	Tissue plasminogen activator
u-PA	Urokinase-type plasminogen Activator
α2-AP	α2-Antiplasmin
