# Supplementary material for: Comparison of Activity and Safety of DSPAα1 and Its N-Glycosylation Mutants
Source: Life (Basel). 2023 Apr 11;13(4):985. doi: 10.3390/life13040985 (PMC10145227; doi:10.3390/life13040985)
Supplement: Supplementary file 1 [file life-13-00985-s001.zip › life-2268137-supplementary.pdf]

life

Article

Supplementary Materials for Comparison of Activity and Safety of DSPAα1 and Its N-Glycosylation Mutants

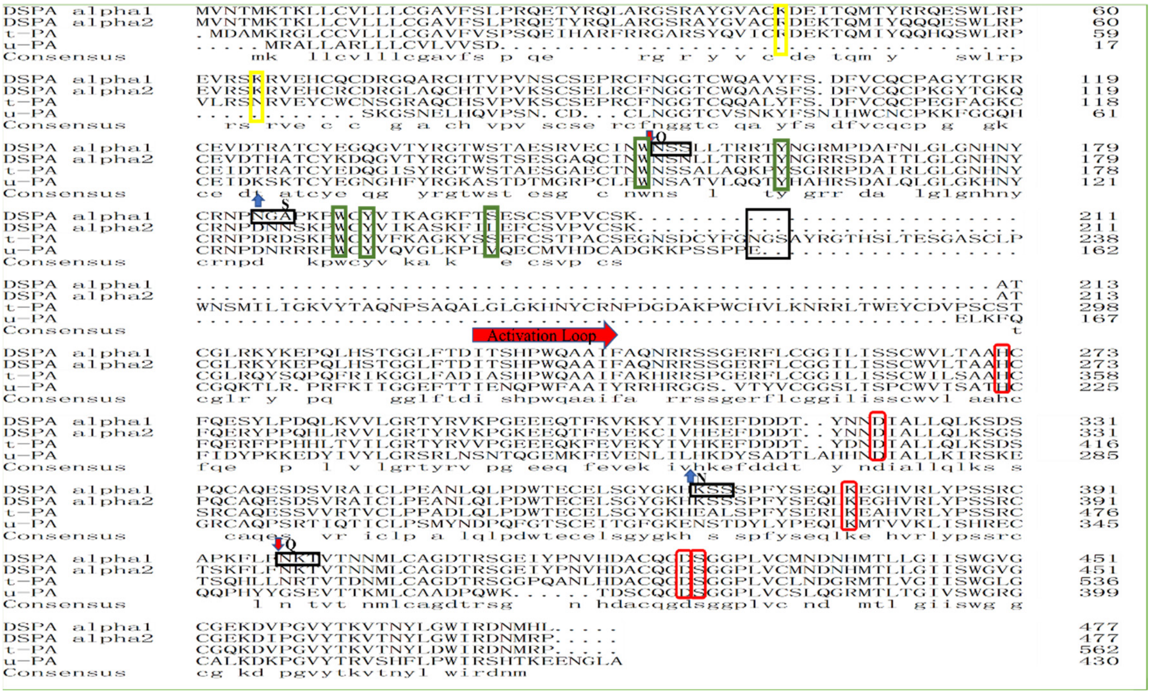

Figure S1. Alignment of animo acid sequences of DSPA α1, DSPA α2, t-PA, and u-PA.

Sequence alignment of DSPAα1 with DSPAα2, t-PA, and u-PA using Clustal. Red rectangular boxes signal the active pocket-associated sites (H272-D321-S428, K379-D427). The thick red arrow represented the activation loop. Yellow rectangular boxes signal the sites associated with the fibrin-sensitive related function in the F region. Green rectangular boxes highlight the ligand binding sites in the K1 region. Black rectangular boxes indicate the glycosylation-related sites. Red arrows pointing downwards represent the de-glycosylation related mutation sites in DSPAα1, and the blue arrows point upwards represent the glycosylation-related mutation sites. The bold letters represent the mutated positions and the mutated amino acid residues.

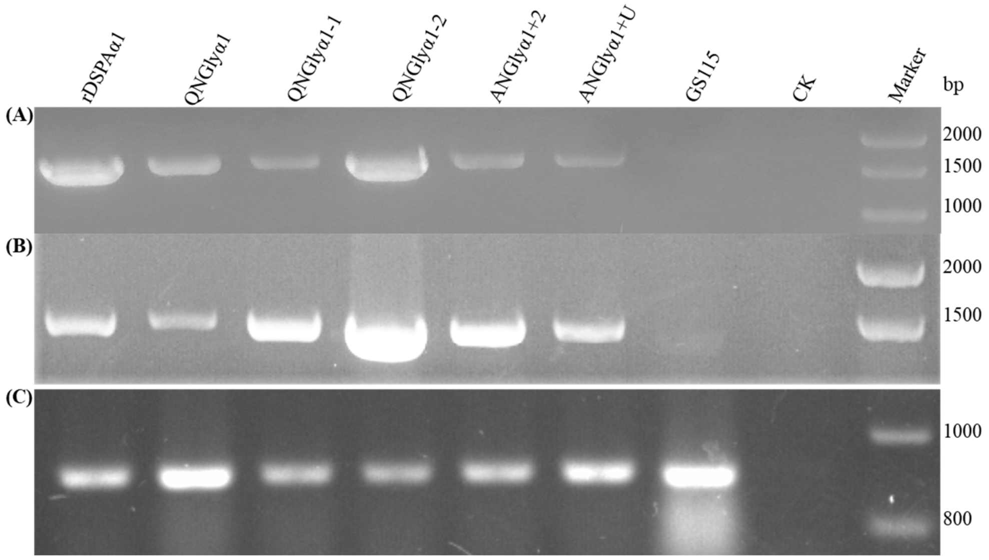

Figure S2. PCR and RT-PCR detection of rDSPAα1 and its mutations.**(A)** The PCR detection of DSPAα1. **(B)** The RT-PCR detection of DSPAα1. **(C)** Actin gene was used as an internal reference control.

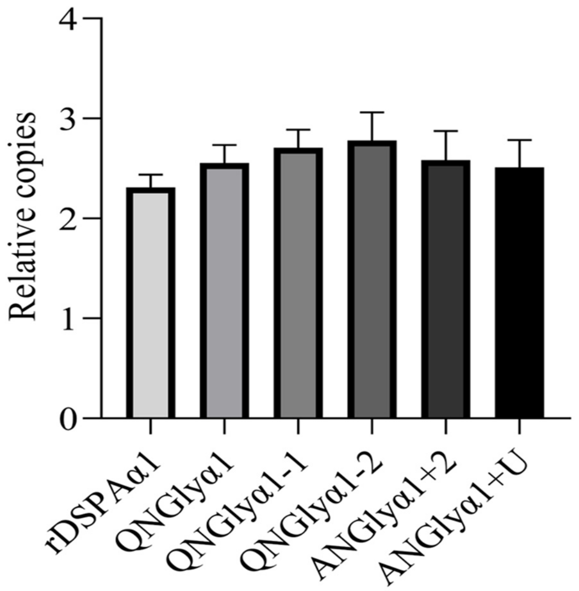

Figure S3. Relative copies of rDSPAα1 and its mutants.All samples showed no significant difference after statistical analysis.

Table S1. the oligonucleotide primers used in this study.

Primers	Sequences
GAP-F	GACGCATGTCATGAGATTATTGGA
ALPHA-F	ATGAGATTTCCATCCATCTTCACTG
DSPAα1-9KY-F	AAGGAGGAGGGTGTTTCCCTCGAGAAAAGAGCCTACGGTGTCGCCTGTAAGGAC GAGAT
DSPAα1His-9KY-R	AGGAACAGTCATGTCTAAGGCGAAATTCGCTTAGTGGTGATGGTGATGGTGCAAG TGCA
QNGlyα1-1-F	CAACTGGCAATCCTCCTTGTTGACCAGAAGAAC
QNGlyα1-1-R	AGGAGGATTGCCAGTTGATACACTCGACTCTGGA
QNGlyα1-2-F	TGTTCCAAAAGACCGTCACCAACAACATGT
QNGlyα1-2-R	CGGTCTTTTGGAACAAGAACTTTGGGGCACA
ANGlyα1+2-F	CTGTAGAAACCCAAACAACAACTCCAAGCCTTGGTGTTACGTCATCAAGG
ANGlyα1+2-R	CGTAACACCAAGGCTTGGAGTTGTTGTTTGGGTTTCTACAGTAGTTGTGG
ANGlyα1+U-F	CGGTTACGGTAAGCACAACTCCTCCTCTCCATTCTACTCC
ANGlyα1+U-R	GGAGTAGAATGGAGAGGAGGAGTTGTGCTTACCGTAACCG
actin-F	AGTGTTCCCATCGGTCGTAG
actin-R	GGTGTGGTGCCAGATCTTTT
actin-Full-F	TCGCTGGTAATCCCGGCTTTTGCTG
actin-Full-R	CTCTACCGGATCCCGCTCGAGGTCG
QF1-F	GTCCTACTTGCCAGACCAAT
QF1-R	ATTGGTCTGGCAAGTAGGAC
QPCRalpha1-F	CGACGACGACACCTACAACA
QPCRalpha1-R	CCGTAACCGGACAACTCACA

Table S2. Protein yields of rDSPAα1 and its mutants after purification.

Samples	Yields (IU in mg of protein/g of Cell Dry Weight)
rDSPAα1	2464.49
QNGlyα1	262.01
QNGlyα1-1	1662.56
QNGlyα1-2	240.26
ANGlyα1+2	2160.40
ANGlyα1+U	2015.19

Table S3. The recanalisation rate of the thrombosis rat model.

Groups	Total numbers	Recanalisation numbers	Recanalisation rate
Mg	8	0	0.00%
rt-PA	8	3	37.50%
rDSPA	8	4	50.00%
QNGly	8	0	0.00%
ANGly	8	3	37.50%
